# Detailed modelling of contact line motion in oscillatory wetting

**DOI:** 10.1038/s41526-021-00186-0

**Published:** 2022-01-19

**Authors:** Gustav Amberg

**Affiliations:** 1 Flow Centre, Department of Engineering Mechanics, The Royal Institute of Technology, 100 44 Stockholm, Sweden; 2 Södertörn University, Alfred Nobels allé 7, 141 89 Huddinge, Sweden

**Keywords:** Chemical engineering, Electrical and electronic engineering

## Abstract

The experimental results of Xia and Steen for the contact line dynamics of a drop placed on a vertically oscillating surface are analyzed by numerical phase field simulations. The concept of contact line mobility or friction is discussed, and an angle-dependent model is formulated. The results of numerical simulations based on this model are compared to the detailed experimental results of Xia and Steen with good general agreement. The total energy input in terms of work done by the oscillating support, and the dissipation at the contact line, are calculated from the simulated results. It is found that the contact line dissipation is almost entirely responsible for the dissipation that sets the amplitude of the response. It is argued that angle-dependent line friction may be a fruitful interpretation of the relations between contact line speed and dynamic contact angle that are often used in practical computational fluid dynamics.

## Introduction

A liquid spreading over a dry surface is a phenomenon that is crucial to many natural processes and important in technology. However, the detailed description and understanding of dynamic wetting is still a complex and challenging problem^1,2^. The fact that the continuum equations of fluid mechanics exhibit a non-integrable singularity^3^ of the viscous stress at the contact line (CL) shows that the detailed microscopic and nanoscopic features of the liquid and the surface will be important for the macroscopic flow. This introduces a host of different processes and phenomena that need to be understood to predict and control wetting processes.

In technology, in addition to such examples as spray painting, coating, etc., one particularly important field is microfluidics^4^. A common challenge is to handle small volumes of liquid, often in the form of small droplets, and one means for achieving this is to use wetting phenomena. Another area where surface tension and wetting become dominant is microgravity^5^. In the absence of gravity, surface tension becomes dominant, and wetting will be important in any fluid handling, from liquid fuel to many daily activities and needs of the astronauts.

Dynamic wetting driven by vibration is both of practical importance and a convenient way to study the phenomenon. The dynamic wetting on a glass plate dipping into a tank and oscillated vertically was studied in ref. ^6^, and damping of surface waves in a rectangular tank was investigated in ref. ^7^, revealing complex dependencies of damping rates on oscillation amplitude. The dynamics of a sessile droplet on a vertically oscillating surface will be sensitive to the detailed conditions at the CL, such as the presence of hysteresis or CL dissipation^8–10^. Xia and Steen^9^ made careful experiments using droplets on a polydimethylsiloxane (PDMS)-covered substrate, which was oscillated vertically at frequencies near drop resonance. The resulting dynamics was examined through phase plots of the dynamic contact angle, the CL position, and the CL speed. In particular, Xia and Steen used this information to measure the CL mobility.

On earth, the size of droplets that can be used is limited to a radius in the order of millimeters, but in microgravity, a larger parameter space can be investigated. In microgravity a droplet size in the order of centimeters can be used instead, which will be advantageous in several respects; one is that spatial dimensions are larger, and the resonance frequency much lower, allowing for higher both spatial and temporal resolution. The larger droplet size also implies that the droplet and CL dynamics are even more dominated by inertia than in a millimeter-sized droplet on earth. It was the intention of Paul Steen to perform such experiments^11^, and these have now been carried out.

From a strictly thermodynamic point of view, a moving CL should be associated with energetic losses of some kind^12^. The idea of a localized dissipation at the CL has been invoked in different ways over the years. Following Hocking^13^, Xia and Steen introduce the concept of a CL mobility *M* as a phenomenological parameter that relates the deviation of the dynamic contact angle from equilibrium *θ* − *θ*_e_ to the CL speed *U*_CL_,

1 $$U_{{{{\rm{CL}}}}} = M\left( {\theta - \theta _{{{\rm{e}}}}} \right),$$

see also refs. ^8,13^. In computational fluid dynamics, more elaborate phenomenological relations between contact angle and CL speed have been devised^14^, which take the form $\theta = f(U_{{{{\rm{CL}}}}},\theta _{{{\rm{e}}}},..)$.

In Molecular Kinetic Theory (MKT)^15,16^, dynamic wetting is described as an activated process on the molecular scale, and the line friction *ζ* is given a phenomenological interpretation on the molecular scale. In its simplest linearized form, this can be written as

2 $$U_{{{{\rm{CL}}}}} = \left( {\gamma {{{{{\mathrm{/}}}}}}\zeta } \right)\left( {{{{\rm{cos}}}}\theta _{{{\rm{e}}}} - {{{{{\mathrm{cos}}}}}}\theta } \right) \approx {{{\rm{sin}}}}\theta _{{{\rm{e}}}}\left( {\frac{\gamma }{\zeta }} \right)\left( {\theta - \theta _{{{\rm{e}}}}} \right),$$

where *γ* is the surface tension and *ζ* is the coefficient of wetting-line friction, which in MKT is estimated in terms of molecular quantities and thermal fluctuations.

In the phase field method, the fluid is viewed as a mixture of two immiscible species. The governing equations are derived from the thermodynamic potentials of such a system to yield typically the Cahn–Hilliard equations^17,18^. The interface now becomes a diffuse region separating the two species, which has a definite width *ε*. The line friction appears as an energy dissipation associated with the CL displacement. Yue and Feng^18^ derive the resulting equivalent condition relating CL speed and dynamic contact angle as

3 $$U_{{{{\rm{CL}}}}} = \left( {\frac{\gamma }{{\left( {\frac{{2\sqrt 2 }}{{3{{{{{\mathrm{{\Gamma}}}}}}}\varepsilon }}} \right)}}} \right)\frac{{{{{\rm{cos}}}}\theta _{{{\rm{e}}}} - {{{\rm{cos}}}}\theta }}{{{{{\rm{sin}}}}\theta }} \approx \left( {\frac{\gamma }{{\left( {\frac{{2\sqrt 2 }}{{3{{{{{\mathrm{{\Gamma}}}}}}}\varepsilon }}} \right)}}} \right)\left( {\theta - \theta _{{{\rm{e}}}}} \right)$$

here Γ is introduced as a rate parameter in the relaxation of the dynamic contact angle boundary condition.

It is noted that the parameters in Eqs. (1)–(3) in the approximation of $\theta - \theta _{{{\rm{e}}}} \ll 1$ can be identified by setting

4 $$M = {{{\rm{sin}}}}\theta _{{{\rm{e}}}}\frac{\gamma }{\zeta } = \frac{\gamma }{{\left( {\frac{{2\sqrt 2 }}{{3{{{{{\mathrm{{\Gamma}}}}}}}\varepsilon }}} \right)}} = \frac{\gamma }{{\mu _f}}$$

In the last equality, we have introduced the line friction *μ*_*f*_, which is essentially the same as the coefficient of wetting-line friction used in ref. ^16^, except that it also absorbs the factor sin*θ*_e_. We note that *ζ* and *μ*_*f*_ have the dimensions of viscosity and that *γ*/*μ*_*f*_ is a velocity.

In many classical treatments, notably the Cox–Voinov law^2^, it is presumed that the static contact angle applies right at the solid boundary and that the angle variations with the speed that are often observed are an “apparent” contact angle, which is attained a short distance away from the wall. It is also often assumed that there is a fluid slip on the wall at the CL, which helps regularize the singularity in stresses. In MKT, and inherent in the introduction of a CL mobility, the contact angle is assumed to be different from the static value right at the wall, when viewed on molecular length scales. In the phase field model, mass diffusion will help regularize the CL and there is no need for a fluid slip. The introduction of dissipation related to CL movement will cause the contact angle at the wall to deviate from the static value.

It is far from clear what the actual conditions are for a given liquid spreading on a particular surface. For a system of decane spreading on a surface covered with a thin layer of PDMS, it was demonstrated experimentally^19^ that the microscopic dynamic contact angle is velocity dependent and deviates substantially from the equilibrium value also at very small but nonzero capillary numbers. In ref. ^20^, a theoretical model is developed that links the distribution of assumed nanoscopic geometrical surface defects to the line friction dissipation. Recent molecular dynamics (MD) simulations have also shown that for water molecules and a wall with hydrogen bonds with the water molecules, the first layer of water molecules are effectively bound to the surface, a no-slip condition is appropriate, and the contact angle deviates from the equilibrium value^21,22^. It is known that the local molecular arrangements will be different in electrowetting and that this will alter the line friction^23,24^. Also, a microscopic geometrical structure on the surface will change the dynamic wetting and can be described through an effective line friction^25^. In an oil–water system, Rondepierre et al.^26^ demonstrated that CL friction was responsible for a decrease in CL speed of three orders of magnitude, as a certain surfactant was added.

The actual nanoscopic cause of local dissipation at the CL can thus be very different depending on the properties of the surface, surface roughness and structure, the liquid properties, the surface chemistry of the wet surface, etc. We conclude that we should not expect any universal answer to the question of what is causing CL friction. Many different nanoscopic or microscopic processes can no doubt have this effect. However, whatever the origin, the effect can be described as a single parameter, the CL friction.

So far, the dependence of line friction on the contact angle has received limited attention, but there is clearly every reason for line friction to vary with the dynamic contact angle. The nonlinear MKT theory gives one possibility and based on MD simulations Johansson and Hess^22^ formulated an expression for the angle dependence of line friction for water molecules on a surface with hydrogen bonds to the water. Other surfaces and liquids may certainly show different behavior.

On this note, we evaluate the detailed experimental results of Xia and Steen^9^, using phase field simulations, with the aim of determining precisely what is required in the mathematical model, in order to faithfully reproduce the experiments. We will study how the angle dependence of the CL friction should be chosen. The model and the simulation are then used to draw conclusions on the source of the dissipation that is evident in the experiment.

## Methods

The simulations are made using the Navier–Stokes–Cahn–Hilliard equations^18,27,28^. These describe the two-phase system as two immiscible species and are motivated by the thermodynamics of such a mixture. A phase field variable is introduced that has different values in the two species, and the fluid interface is identified as the steep but continuous transition between those values.

5 $$\rho \frac{{D{{{{{\mathbf{u}}}}}}}}{{Dt}} = - \nabla S + \mu \nabla ^2{{{{{\mathbf{u}}}}}} - C\nabla \phi + \rho \ddot a{{{{{\mathbf{e}}}}}}_{{{{{\boldsymbol{z}}}}}}$$

6 $$\nabla \cdot {{{{{\mathbf{u}}}}}} = 0$$

7 $$\frac{{DC}}{{Dt}} = \kappa \nabla ^2\phi$$

8 $$\phi = \frac{\sigma }{\varepsilon }f^\prime \left( C \right) - \sigma \varepsilon \nabla ^2C$$

with the standard choices: $f\left( C \right) = \left( {1 - C} \right)^2\left( {1 + C} \right)^2{{{{{\mathrm{/}}}}}}4$, and $g\left( C \right) = \left( {2 + 3C - C^3} \right){{{{{\mathrm{/}}}}}}4$.

Here **u**, *C*, and *ϕ* are the fluid velocity, the phase field variable, and the associated chemical potential, respectively. *C* is +1 in the liquid and −1 outside the droplet. *S* is a reduced pressure such that $p = S + C\phi$ is the actual pressure. *μ* and *ρ* are the local viscosity and density, respectively, and *κ* is the phase field mobility. *σ* and *ε* are the phase field parameters, where *ε* denotes the interface thickness, and *σ* gives the surface tension *γ* through $\gamma = \sigma ( {2\surd 2} ){{{{{\mathrm{/}}}}}}3$. The function $f\left( C \right) = \left( {1 - C} \right)^2\left( {1 + C} \right)^2{{{{{\mathrm{/}}}}}}4$ represents the standard choice in phase field methods and gives a qualitatively reasonable thermodynamic behavior representative of an immiscible mixture.

The simulations are performed in a cylindrical coordinate system that follows the oscillating substrate, giving rise to the acceleration term on the right-hand side of the momentum equation, with $a\left( t \right) = A_{{{\rm{d}}}}\sin 2\pi ft$ denoting the vertical position of the substrate.

The boundary conditions on the solid wall express that the fluid cannot penetrate through the wall and does not slip on the wall.

9 $$\nabla \phi \cdot {{{{{\boldsymbol{n}}}}}} = 0$$

10 $${{{{{\boldsymbol{u}}}}}}_s = 0$$

One additional boundary condition is needed for the phase field variable, which expresses the wetting conditions.

11 $$\varepsilon \mu _f\frac{{\partial C}}{{\partial t}} = - \sigma \varepsilon \nabla C \cdot {{{{{\boldsymbol{n}}}}}} + \left( {\gamma _1 - \gamma _0} \right)g^\prime \left( C \right)$$

Here *σ* and *ε* are the phase field parameters as above, giving surface tension as $\gamma = \sigma ( {2\surd 2} ){{{{{\mathrm{/}}}}}}3$. *γ*_1_ and *γ*_0_ are the surface energies of the dry and wet solid surface, respectively, so that the equilibrium contact angle *θ*_e_ is given by $\left( {\gamma _1 - \gamma _0} \right){{{{{\mathrm{/}}}}}}\gamma = \cos \theta _{{{\rm{e}}}}$. The form of the function $g\left( C \right) = \left( {2 + 3C - C^3} \right){{{{{\mathrm{/}}}}}}4$ is chosen in relation to the function *f* in Eq. (4).

Yue^29^ developed a phase field treatment of contact angle hysteresis, where advancing and receding contact angels are introduced in a piecewise continuous function on the right-hand side of the equation corresponding to Eq. (11). The CL is then allowed to move according to whether the value of the dynamic contact angle exceeds (subceeds) the advancing (receding) angle. A treatment inspired by this is also developed for level-set methods^30^.

The left-hand side represents the dissipation associated with CL motion, quantified by the parameter *μ*_*f*_, which we will call CL friction. This has dimensions of viscosity.

By considering solutions to the Cahn–Hilliard equations near equilibrium at the CL, an explicit relation between CL speed *U*_CL_ and dynamic contact angle *θ* can be derived from Eq. (11), see ref. ^18^ and Eq. (3):

12 $$\frac{{\mu _{{{\rm{f}}}}U_{{{{\rm{CL}}}}}}}{\gamma } = \frac{{{{{\rm{cos}}}}\theta _{{{\rm{e}}}} - {{{\rm{cos}}}}\theta }}{{{{{\rm{sin}}}}\theta }} \approx \theta - \theta _{{{\rm{e}}}}$$

The dynamic contact angle is equal to the equilibrium angle if *μ*_*f*_ = 0, in which case there is also no dissipation at the CL. The last approximate equality holds if *θ* is near *θ*_e_.

The above equations are made nondimensional using *R* as length reference, the radius of the half-sphere (approximately the initial wet footprint radius), and an inertial capillary velocity $U = \sqrt {\gamma {{{{{\mathrm{/}}}}}}\left( {\rho R} \right)}$.

The nondimensional parameters that appear are

**Table:** 

${{{\rm{Oh}}}} = \frac{\mu }{{\sqrt {\rho \gamma R} }}$	Ohnesorge number
${{{\rm{Oh}}}}_f = \frac{{\mu _f}}{{\sqrt {\rho \gamma R} }}$	Line friction Ohnesorge number
${{{\rm{Bo}}}} = \frac{{\rho gR^2}}{\gamma }$	Bond number, $g = 9.81\,{{{\rm{m}}}}{{{{{\mathrm{/}}}}}}{{{\rm{s}}}}^2 < ?tpb 5pt? >$
$\omega = 2\pi f\sqrt {\frac{{\rho R^3}}{\gamma }}$	Nondimensional oscillation angular frequency. *f* is the oscillation frequency in Hertz
*A* = *A*_d_/*R*	Nondimensional oscillation amplitude

In addition to these, the Cahn–Hilliard equations use the following parameters:

**Table:** 

${{{\rm{Pe}}}} = \frac{{UR}}{D} = \sqrt {\frac{\gamma }{{\rho R}}} \frac{R}{{\frac{{2\sqrt 2 }}{3}\frac{{\kappa \gamma }}{\varepsilon }}}$	Peclet number related to phase field mobility. Set to 100 in the simulations. Reported here
${{{\rm{Cn}}}} = \frac{\varepsilon }{R}$	Cahn number, nondimensional interface width. Kept at 0.01 in the simulations. Reported here
$\frac{{\rho _{{{{\rm{air}}}}}}}{{\rho _{{{{\rm{liq}}}}}}}$	Ratio between air and liquid density. Set to 0.01 here, for computational convenience
$\frac{{\mu _{{{{\rm{air}}}}}}}{{\mu _{{{{\rm{liq}}}}}}}$	Ratio between air and liquid viscosity. Set to 0.03 here, for computational convenience

The simulations are carried out using an adaptive finite element solver, as in ref. ^31^. The adaptive grid is automatically refined as needed down to an element size of 0.001. The air viscosity and density are taken larger than those of air but much smaller than the values for the liquid. They are deemed small enough for the air to have a negligible influence on the flow inside the droplet.

### Reporting summary

Further information on research design is available in the Nature Research Reporting Summary linked to this article.

## Results

### Flow fields

Xia and Steen report the most detailed results for their experiment labeled M00, using a 20 µl water droplet oscillated at *f* = 61 Hz, with an amplitude of *A*_d_ = 0.1 mm. The substrate is a slightly hydrophobic PDMS-covered silicon surface, with a static contact angle of *θ*_e_ = 101°. This will be the reference case for the simulations shown here.

The nondimensional numbers for this case are Oh = 0.00256, amplitude *A* = 0.047, and angular velocity of the driving *ω* = 4.41. The low value of the Ohnesorge number indicates that the droplet dynamics are inertial and that viscosity plays a minor role. Near the solid surface, we should expect a viscous Stokes layer of thickness $\sim \sqrt {\mu {{{{{\mathrm{/}}}}}}(\rho \pi f)}$, which is approximately 0.07 mm for the experimental parameters, i.e., much less than the initial droplet radius of 2.1 mm.

The order unity value of the angular velocity shows that the oscillations are reasonably matched with the inertial timescale, as is expected since the experiment aims at being near resonance. The value of the line friction Ohnesorge number Oh_*f*_ is set to unity, which makes the reference value of the line friction $\mu _{f,{{{\rm{ref}}}}} = \sqrt {\rho \gamma R}$. We will return to the more detailed modeling of the line friction below.

**Fig. 1:**
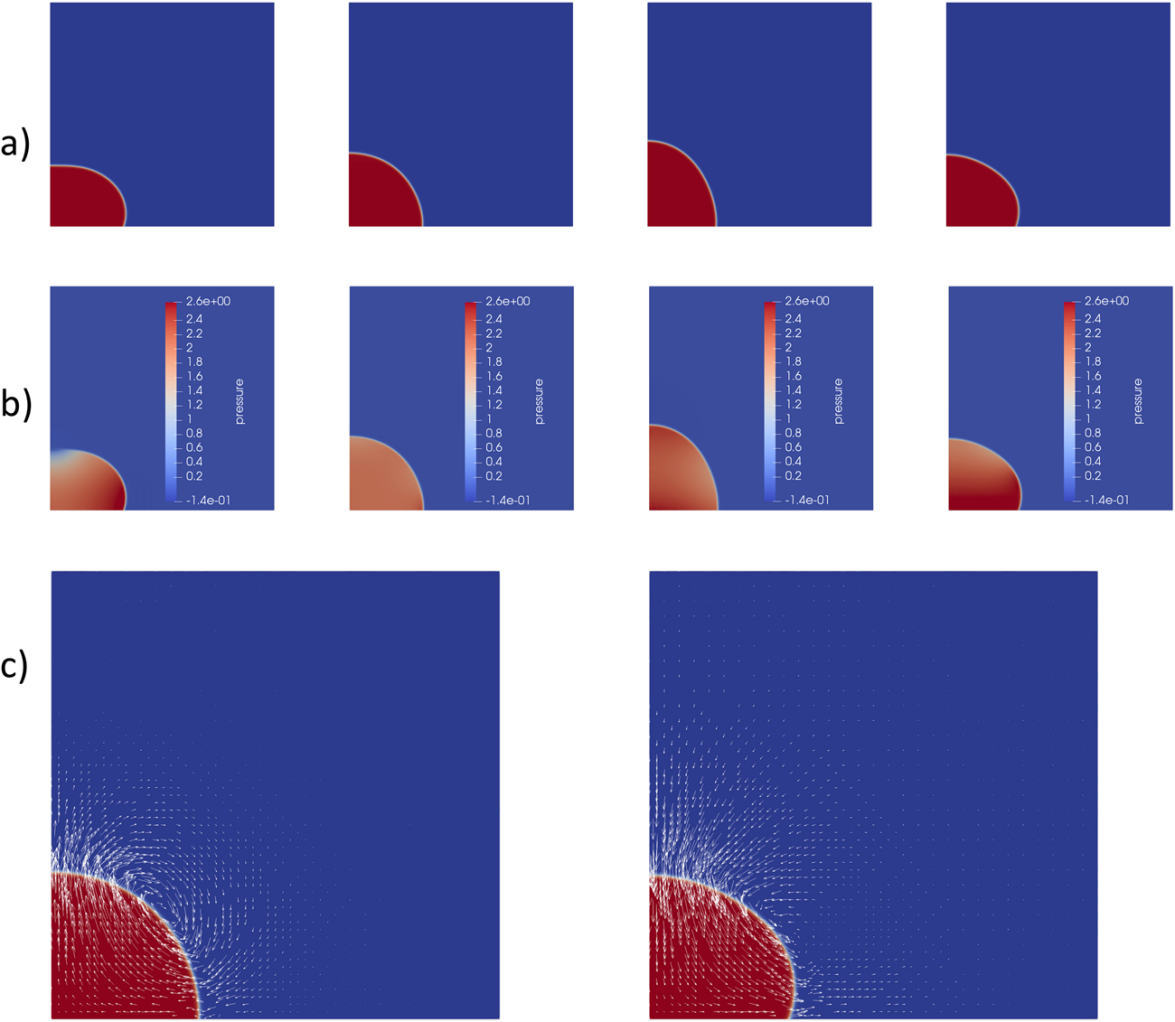
One oscillation cycle for the M00 case. **a** The shape of the droplet at four instants. **b** The pressure field. **c** Velocity field at the instants corresponding to frame two and four of **a** and **b**.

Figure 1 shows the simulation results over one cycle for the M00 case. In Fig. 1a, the first frame shows the position near the lower turning point when the droplet is compressed towards the substrate. In the second frame, it is extending upwards and is near its most elongated state in the third frame. In the fourth frame, it is being again compressed as the substrate is moving upwards.

A Weber number based on the oscillation amplitude *A*_d_ and frequency *f* can be expressed as *A*^2^*ω*^2^, giving the value $0.047^24.41^2 = 0.043$. We thus expect the flow to be dominated by the surface tension.

Figure 1b shows the corresponding pressure fields, always reflecting the curvature of the interface. The pressure is elevated in the droplet due to the capillary pressure and fluctuates as the interface curvature varies. In the most compressed state in the first frame of Fig. 1b, there is a concave shape at the top of the droplet, which causes a local low pressure there. The pressure in the air is almost constant, due to the low air density. Figure 1c shows the velocity fields for the points where the droplet is extending upwards and being compressed (corresponding to frames two and four of Fig. 1a, b).

**Fig. 2:**
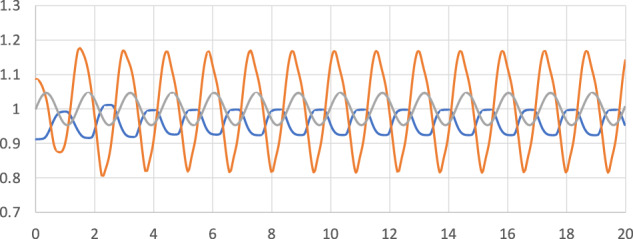
Time signals for the contact line position *r*_CL_ (blue), the droplet height *z* (yellow), and the substrate position *A*sin*ωt* (gray). The substrate position is drawn around an equilibrium position of 1, to facilitate comparison with the CL position and the droplet height.

In Fig. 2 are shown the time signals for the vertical substrate position, together with the CL position and the droplet height. It is noticed that the amplitude of the droplet height is about three times that of the substrate amplitude, as expected for a near resonance situation. All signals quickly go into a periodic motion, with no sign of period-doubling or other more complex dynamics. The responses in both the droplet height and the CL position are nonlinear, however, with shapes departing from sinusoidal.

The CL position signal deviates from a sinusoidal shape, and tends towards a square wave, with flat peaks. This is a signature of a stick-slip motion of the CL; it is rather stationary at its extreme values but shifts quickly between them twice per cycle. We will see that this is built into the simulation through angle-dependent line friction.

### Model for angle-dependent line friction

In addition to the input data already discussed, angle-dependent line friction has been implemented in this simulation. The line friction in Eqs. (11) and (12) is computed from a regularized well function:

13 $$\mu _f = \mu _{f,{{{\rm{ref}}}}}\mu _f^ \ast \left( \theta \right)$$

where the nondimensional $\mu _f^ \ast \left( \theta \right)$ is

14 $$\mu _f^ \ast \left( \theta \right) = \mu _{f0}\left( {1 - H\left( \theta \right)} \right) + \mu _{f1}H\left( \theta \right)$$

and

15 $$H\left( \theta \right) = 1 + 1/2\left(\tanh \left( {\frac{{\theta - \left( {\theta _{{{\rm{e}}}} + {{{\rm{d}}}}\theta } \right)}}{\delta }} \right) - \tanh \left( {\frac{{\theta - \left( {\theta _{{{\rm{e}}}} - {{{\rm{d}}}}\theta } \right)}}{\delta }} \right)\right)$$

**Fig. 3:**
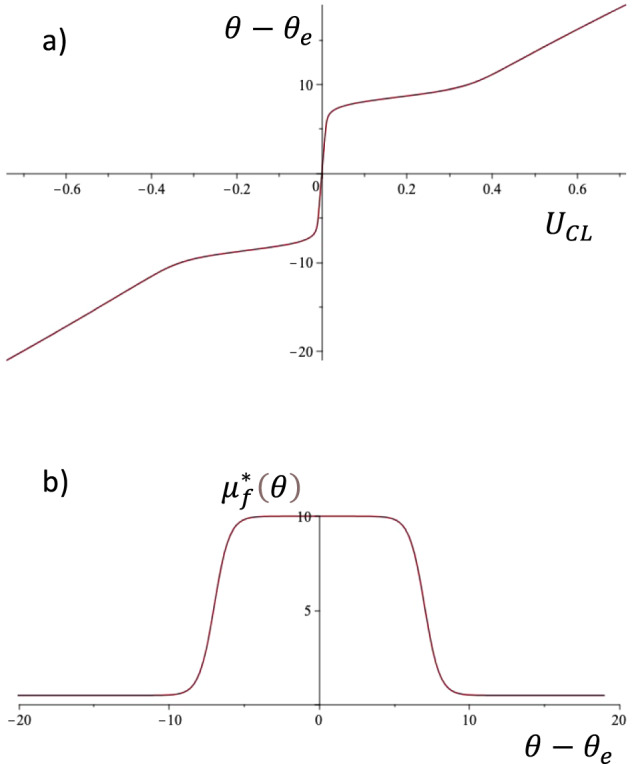
Model of angle-dependent line friction, according to Eqs. (12)–(15). **a** The deviation of the dynamic contact angle from the equilibrium angle *θ* − *θ*_e_ as a function of contact line speed. **b** Nondimensional line friction as a function of *θ* − *θ*_e_. Parameter values are the same as for the simulations in Figs. 1, 2, and 4: *θ*_e_ = 101°, *dθ* = 7°, *δ* = 1°, *μ*_*f*0_ = 10, and *μ*_*f*1_ = 0.5.

**Fig. 4:**
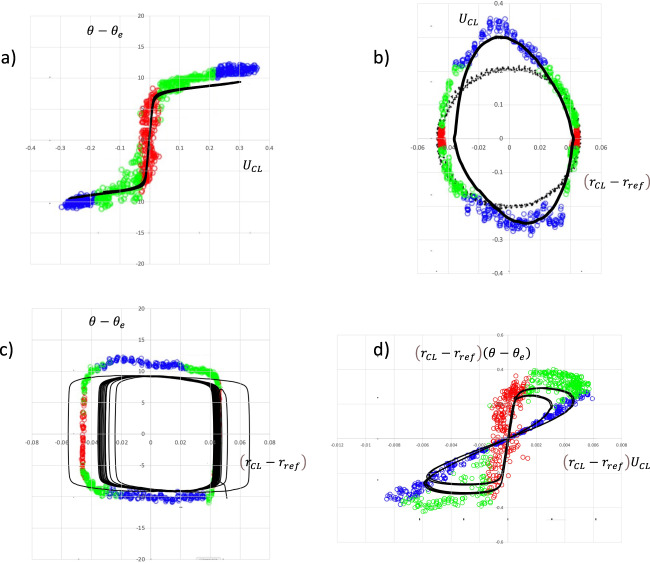
Comparison of phase plots with Xia and Steen experiments for the case M00. Colored dots are the experimental results of Xia and Steen, and the black solid curves are the present simulations. **a** Dynamic contact angle departure from the equilibrium angle *θ* − *θ*_e_ as a function of contact line speed. **b** Contact line speed vs contact line position, as a departure from the mean position. **c** Dynamic contact angle *θ* − *θ*_e_ vs contact line position. **d** Contact line speed vs contact line position, as a departure from the mean position. Dynamic contact angle departure from the equilibrium angle multiplied by contact line position $\left( {r_{{{{\rm{CL}}}}} - r_{{{{\rm{ref}}}}}} \right)\left( {\theta - \theta _{{{\rm{e}}}}} \right)$ vs contact line position multiplied by contact line speed $\left( {r_{{{{\rm{CL}}}}} - r_{{{{\rm{ref}}}}}} \right)U_{{{{\rm{CL}}}}}$.

**Fig. 5:**
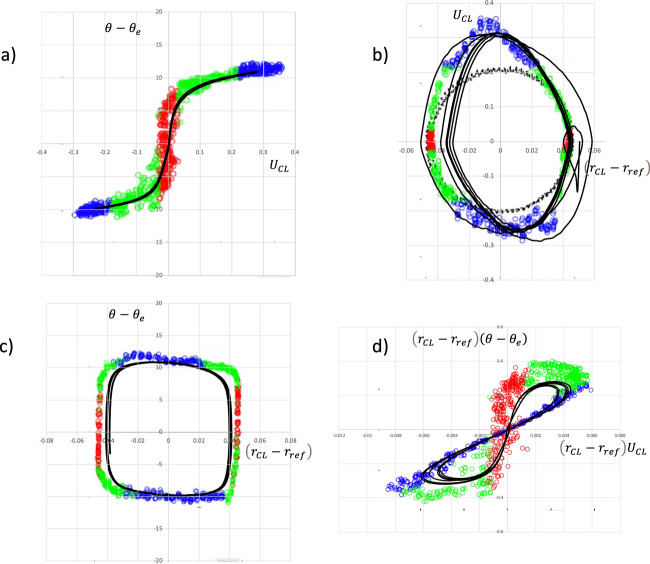
Comparison of phase plots with Xia and Steen experiments for the case M00, using a closer fit for the TD. Colored dots are the experimental results of Xia and Steen, and the black solid curves are simulations. **a** Dynamic contact angle departure from the equilibrium angle *θ* − *θ*_e_ as a function of contact line speed. **b** Contact line speed vs contact line position, as a departure from the mean position. **c** Dynamic contact angle *θ* − *θ*_e_ vs contact line position. **d** Contact line speed vs contact line position, as a departure from the mean position. Dynamic contact angle departure from the equilibrium angle multiplied by contact line position $\left( {r_{{{{\rm{CL}}}}} - r_{{{{\rm{ref}}}}}} \right)\left( {\theta - \theta _{{{\rm{e}}}}} \right)$ vs contact line position multiplied by contact line speed $\left( {r_{{{{\rm{CL}}}}} - r_{{{{\rm{ref}}}}}} \right)U_{{{{\rm{CL}}}}}$.

**Fig. 6:**
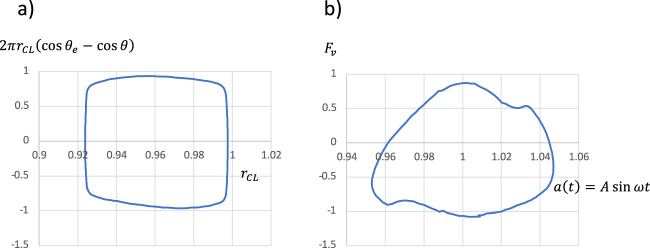
Calculation of CL dissipation. **a** Uncompensated Young’s stress $2\pi r_{{{{\rm{CL}}}}}\left( {\cos \theta _{{{\rm{e}}}} - \cos \theta } \right)$ vs *r*_CL_ over one cycle. The area inside the loop is 0.131779. **b** Vertical force from the substrate on the drop vs substrate displacement $a\left( t \right) = A\sin \omega t$. The area inside the loop is 0.132914.

Equation (14) thus describes nondimensional line friction that has a high value *μf*_0_ in the sticking region in the angle range *θ*_e_ ± d*θ*, and a low value *μf*_1_ outside of this range. *δ* smooths the corners of the well function and *μ*_*f*,ref_ is a dimensional reference value that is used in the line friction Ohnesorge number Oh_*f*_. In the present case, Oh_*f*_ = 1, and thus $\mu _{f,{{{\rm{ref}}}}} = \sqrt {\rho \gamma R}$. In the simulation in Figs. 1–6, these values are: *θ*_e_ = 101°, d*θ* = 7°, *δ* = 1°, *μf*_0_ = 10, *μf*_1_ = 0.5 and Oh_*f*_ = 1. We could interpret *θ*_e_ + d*θ* = *θ*_a_ as approximating the advancing contact angle and *θ*_e_ − d*θ* = *θ*_r_ as the receding angle.

Inserting the line friction according to Eq. (14) into Eq. (12), we readily obtain the dynamic contact angle as a function of CL speed (see Fig. 3). As expected, the high line friction near the equilibrium angle creates a region of near stick, with low velocities for angles in a region ≈±7° around the equilibrium. In this region, the well function *H*(*θ*) ≈ 0, and $\mu _f^ \ast \left( \theta \right)$ will be constant and equal to the high value *μ*_*f*0_. For CL speeds up to about 0.3, the angle is approximately constant, between 7 and 10 degrees, establishing a “slip” region. For angles departing >10 degrees from equilibrium, or speeds larger than approximately 0.4, the well function *H*(*θ*) is 1 and the line friction $\mu _f^ \ast \left( \theta \right)$ becomes equal to the low value *μ*_*f*1_. To summarize: in the window $\left| {\theta - \theta _{{{\rm{e}}}}} \right| \, < \, {{{\rm{d}}}}\theta$, the line friction is constant, equal to the high value *μ*_*f*0_, and for angles distinctly outside this window, $\left| {\theta - \theta _{{{\rm{e}}}}} \right| \, > \, {{{\rm{d}}}}\theta$, the line friction tends to the constant value *μ*_*f*1_. Connecting these two regions is a velocity range where the dynamic angle is approximately constant, i.e. a region of “free slip”.

As seen in Fig. 3a, there is still some variation of angle with CL speed also in the region of “free slip”. The slope of the curve in Fig. 3a in this region is related to *δ*, the width of the transition region in line friction in Fig. 3b, and the line friction far from equilibrium *μ*_*f*1_. Approximating the contact angles and the CL speeds at the ends of the transition region as $\theta _0 = \theta _{{{\rm{e}}}} + {{{\rm{d}}}}\theta + \delta$, $\theta _1 = \theta _{{{\rm{e}}}} + {{{\rm{d}}}}\theta - \delta$, and $U_0 = (\theta _0 - \theta _{{{\rm{e}}}}){{{{{\mathrm{/}}}}}}\mu _{f0}$, $U_1 = (\theta _1 - \theta _{{{\rm{e}}}}){{{{{\mathrm{/}}}}}}\mu _{f1}$, and assuming that $\mu _{f1} \ll \mu _{f0}$, an estimate of the slope in Fig. 3a in the transition or “free slip” region can be obtained as ${\Delta}\theta {{{{{\mathrm{/}}}}}}{\Delta}{{{{{\mathrm{U}}}}}} \approx 2\mu _{f1}{{{{{\mathrm{/}}}}}}\left( {1 + \frac{{{{{\rm{d}}}}\theta }}{\delta }} \right)$ (angles in radians). For a very steep transition region ($\delta \ll {{{\rm{d}}}}\theta$) the slope will thus approach zero, while it becomes comparable to *μ*_*f*1_ for *δ* ~ d*θ*.

The model for line friction in Eqs. (13)–(15) was constructed to be as conceptually simple as possible, characterized by a high friction region around equilibrium, and low friction further from equilibrium. In order to test this against the experiments of Xia and Steen the simulated results are overlaid on their experimental phase plots (their Figs. 4 and 5), see Fig. 4.

Figure 4a shows the graph of dynamic contact angle vs CL speed, the “traditional diagram” (TD) as it is referred to by Xia and Steen. The colored circles are the experiments of Xia and Steen, and the black curve is obtained from the numerical simulation. If the CL friction would be a constant this curve would always be a straight line, thus the angle variation in the CL friction becomes important. The parameters d*θ*, *δ*, *μ*_*f*0_, and *μ*_*f*1_ for the angle-dependent line friction are chosen to give a fair approximation of the TD, in the following manner. d*θ* is chosen to capture the magnitude of the sticking region in Fig. 4a, and the sticking region line friction, *μ*_*f*0_, sets the slope of the curve at the origin in Fig. 4a. The transition region width *δ* allows for the smooth curvature of the TD at the end of the sticking region. The line friction far from equilibrium *μ*_*f*1_ is then chosen to approximate the slope of the TD in the “free slip” region. It should be noted that the numerical simulation accurately reproduces the theoretical curve in Fig. 3a and that the CL velocities encountered in the experiment are always below about 0.3, so that only the “sticking” and the “free slip” regions are visited.

Figure 4b shows a phase plot in terms of the CL speed vs the CL position. In addition to the experimental data shown as colored circles, the black circles denote the position of the substrate. The black line is the trajectory from the simulation. It captures both the elongation and the slight inclination of the experimental loop. The width of the simulated trajectory is slightly less than the experimental one, but overall, the agreement is good.

Figure 4c shows the dynamic contact angle vs CL position. Here the stick–slip character of the motions is evident; the shape of the trajectory is a quadrilateral, where the horizontal upper and lower parts show the rapid phase when the CL moves from one almost steady position to another, at a fairly constant contact angle. The vertical sides represent the “stick” phase where the CL is nearly stationary and the angle changes.

Figure 4d was introduced by Xia and Steen to highlight the “stick” and the “slip” parts of the motions and quantify those separately. The graph shows dynamic contact angle departure from the equilibrium angle multiplied by CL position $\left( {r_{{{{\rm{CL}}}}} - r_{{{{\rm{ref}}}}}} \right)\left( {\theta - \theta _{{{\rm{e}}}}} \right)$ vs CL position multiplied by CL speed $\left( {r_{{{{\rm{CL}}}}} - r_{{{{\rm{ref}}}}}} \right)U_{{{{\rm{CL}}}}}$. The nonlinearity that is introduced creates two loops, one for the receding and one for the advancing motion of the CL. The simulated trajectory is again in fair agreement with the experiment, even though the experiment extends somewhat further from the origin. In view of the scatter in the experimental data the agreement in Fig. 4 was deemed sufficient for the present discussion.

In terms of the model in Eqs. (13)–(15), we see that the sticking phase is characterized by a high constant line friction *μ*_*f*0_ = 10. This shows up in the central part of Fig. 4d, where the simulated trajectory is steep and near the red experimental circles. The slope of the curve here is proportional to *μ*_*f*0_. The main source of CL dissipation is at the slip phase, as the uncompensated Young’s stress at the receding or advancing contact angle multiplies the CL speed. Here the CL slip speed becomes rather independent of the dynamic angle. In a macroscopic experiment where the overall CL speed is measured along with the contact angle, an overall CL friction for an advancing CL would be identified from Eq. (12) as $\mu _f = \left( {\gamma {{{{{\mathrm{/}}}}}}U_{{{{\rm{CL}}}}}} \right)\left( {{{{\rm{cos}}}}\theta _{{{\rm{e}}}} - {{{\rm{cos}}}}\theta _{{{\rm{a}}}}} \right){{{{{\mathrm{/}}}}}}{{{\rm{sin}}}}\theta _{{{\rm{a}}}}$. This would not be possible to directly relate to material parameters, since *U*_CL_ here would be determined from the overall droplet dynamics, and the angle would stay nearly constant, close to the advancing angle. To predict the wetting behavior, the CL friction would need to be modeled and the overall dynamics simulated. For angles further away from the sticking region, $\mu _f^ \ast \left( \theta \right)$ becomes *μ*_*f*1_ and Eq. (12) reduces to $\left( {\mu _{f1}U_{{{{\rm{CL}}}}}} \right){{{{{\mathrm{/}}}}}}\gamma = \left( {{{{\rm{cos}}}}\theta _{{{\rm{e}}}} - {{{\rm{cos}}}}\theta } \right){{{{{\mathrm{/}}}}}}{{{\rm{sin}}}}\theta$ with a constant line friction. We do not however have any experimental data to verify if this is indeed the case.

The CL mobility parameter *M* (Eq. (1)), as identified by Xia and Steen is the inverse of the slope of the line formed by the blue circles in Fig. 4d. This is also approximately the same as the inverse of the slope of the straight line obtained by connecting the two regions of blue circles in the “wings” of Fig. 4a.

The agreement with the experiment in Fig. 4 is fair, but there are some differences, so more precise modeling of the experimental points in the TD in Fig. 4a was made. The model in Eqs. (13)–(15) is symmetric around the equilibrium angle, while the experimental points show some differences between the advancing and receding curves. A more complex mathematical expression for the angle dependence of the line friction was designed so that the TD is approximated more closely, see Fig. 5a. Overall the agreement in all four panels is somewhat improved but not dramatically so. The conclusion is that the complexity of Eqs. (13)–(15), with the four parameters d*θ*, *δ*, *μ*_*f*0_, and *μ*_*f*1_ is sufficient to capture the essential features of the flow.

### Energy dissipation

It may be asked what the nature is of the dissipation that limits the response amplitude. In the simulation for the standard M00 case, the input energy per cycle was determined from the pressure and speed of the substrate integrated over a cycle. Figure 6b shows a graph of the total vertical force *F*_v_ from the substrate, vs the substrate position over a cycle. In the same manner, the dissipation at the CL was determined from a graph of $2\pi r_{{{{\rm{CL}}}}}\left( {\cos \theta _{{{\rm{e}}}} - \cos \theta } \right)$ vs *r*_CL_ graph, see Fig. 6a.

The total amount of work supplied to the drop by the oscillation over a cycle is now the area inside the loop in Fig. 6b, which is calculated as 0.132914. The area inside the loop in Fig. 6a giving the CL dissipation is 0.131779. As expected, the work input is slightly larger than the CL dissipation, with a relative difference of 0.8%. We should not overinterpret this small difference, in view of other possible sources of inaccuracy, but this still shows that the CL dissipation is the completely dominating cause of damping in this case. There is an almost perfect match between the input energy and the energy dissipated at the CL over a cycle. The other source of dissipation by bulk viscous dissipation is indeed expected to be quite small, given the small value of the Ohnesorge number (Oh = 0.00256).

## Discussion

The good qualitative and quantitative agreement between simulation and experiment, in all the different aspects that can be accessed through the phase plots presented by Xia and Steen, indicates that the essential physical processes are well represented in the mathematical model. The experiment is quite sensitive since the response is amplified through the resonance of the droplet motion, and the damping that is present in the system is controlling the resulting periodic CL motion completely. As shown in Fig. 6, the damping is almost entirely due to the CL dissipation. Simulations were performed where the CL dissipation is removed, either by enforcing the static angle (by setting *μ*_*f*_ = 0) or pinning the CL position. The amplitude of the droplet motion then grows quickly, and the droplet will break within a few periods.

The most important part of the mathematical model is the angle-dependent line friction in Eqs. (13)–(15). This simple function allows for a narrow high friction region near equilibrium, and much lower friction outside this region, and this is sufficient for capturing the essentials of the flow dynamics.

The simulations presented here only show results for an inertial flow with a low Ohnesorge number. It may be asked if the behavior of a viscous drop, with Oh ≥ 1 could be described in a similar way, with hysteresis modeled as angle-dependent line friction. While this has not been carried out here, I expect this to be the case since the line friction is local to the CL and not directly dependent on the internal bulk flow. It should be noted though that the experimental arrangement of Xia and Steen would probably not be suitable for very viscous drops, since the resonance is needed to have a large enough amplitude of the CL motion.

This also exemplifies how empirical models that postulate a relation between CL speed and dynamic contact angle can be interpreted through Eq. (12) as revealing the angle dependence of the line friction. As commented above, the line friction can have many different micro- or nanoscopic causes, and thus may depend on the dynamic angle in different ways. But in attributing the CL variation to the line friction and hypothesizing it to be reflecting processes that are local to the CL, we could hope to decouple it from the macroscopic flow problem. It would then be possible to restrict the problem to analyzing the micro- or nano-specifics of a particular surface and a particular fluid to find the line friction and its angle dependence^22^ and then hope that this line friction could be used with accuracy for all flow situations, for that particular combination of fluid and surface.

## Data Availability

The datasets generated during and/or analyzed during the current study are available from the corresponding author on reasonable request.

## Code availability

The code used in the current study is available from the corresponding author on reasonable request.
